# Early Mention of the Term Epidemiology

**DOI:** 10.3201/eid2211.141466

**Published:** 2016-11

**Authors:** José Tuells

**Affiliations:** University of Alicante, Alicante, Spain

**Keywords:** epidemiology, plague, Quinto Tiberio Angelerio, Joaquín de Villalba, Sardinia

**To the Editor:** The excellent article on measures for controlling plague in Alghero, Sardinia, describes the procedures introduced by the Calabrian doctor Quinto Tiberio Angelerio (1532–1617) to combat an outbreak during 1582–1583 (*1*). The authors cite 2 works published by Angelerio relating to these events, Ectypa (1588) (*2*) and Epidemiologìa (1598) (*3*). To say that Epidemiologìa was written only in Spanish is a small error, however, because both works were written in Latin. Ectypa contains an appendix written in Catalan with the measures to take during an epidemic, whereas in Epidemiologìa, this appendix was written in Spanish.

A third and posthumous edition, not cited in the article, was found recently in the Bibliothèque Nationale de France (*4*). Epydem (*5*) was published in Naples in 1651 by Angelerio’s nephew. This work was written in Latin and did not contain appendices but did include a brief biography of Angelerio. The terms epidemic (Greek) and plague (Latin) were used ambiguously to refer to “maladies that came from abroad or afflicted us collectively.”

The major aspect of Angelerio’s texts, especially Epidemiologìa (*3*), is that the term epidemiology was used here for the first time in a “treatise on the plague” in the sense of “how to protect yourself from it when it erupts.” The term was adopted by the Spanish physician Joaquín de Villalba (1752–1807) who, citing Angelerio, used it as the title for his work Epidemiología Española (*6*). This treatise gained wide circulation, and the term was espoused by various authors from the beginning of the 19th century onward. Villalba used it to compose a historical chronology of the epidemics in Spain, noting the type of disease and the place and year in which it had occurred; this was an initial approach to the concept of epidemiology, **which coincided with the development of medical topographies and statistics applied to infectious diseases**.
